# High-throughput extraction on a dynamic solid phase for low-abundance biomarker isolation from biological samples

**DOI:** 10.1038/s41378-023-00582-4

**Published:** 2023-09-06

**Authors:** Lucile Alexandre, Monica Araya-Farias, Manh-Louis Nguyen, Nikoletta Naoumi, Giacomo Gropplero, Electra Gizeli, Laurent Malaquin, Stéphanie Descroix

**Affiliations:** 1 Laboratoire Physico-Chimie Curie, CNRS UMR 168, Institut Curie, PSL Research University, Paris, France; 2 Institut Pierre-Gilles de Gennes (IPGG), Sorbonne University, Paris, France; 3 Department of Biology, University of Crete, Heraklion, Greece; 4 Institute of Molecular Biology and Biotechnology (IMBB) - FORTH, Heraklion, Greece; 5 Laboratoire d’analyse et d’architecture des systèmes (LAAS) CNRS, Elia Group, Toulouse, France; 6 Present Address: Frédéric Joliot Institute for Life Sciences, Pharmacology and Immunoanalysis Unit, Immunoanalysis Studies and Research Laboratory, Alternative Energies and Atomic Energy Commission (CEA), Gif-sur-Yvette, France

**Keywords:** Engineering, Microfluidics

## Abstract

Liquid biopsy, in particular circulating tumor DNA (ctDNA) analysis, has paved the way for a new noninvasive approach to cancer diagnosis, treatment selection and follow-up. As a crucial step in the analysis, the extraction of the genetic material from a complex matrix needs to meet specific requirements such as high specificity and low loss of target. Here, we developed a new generation of microfluidic fluidized beds (FBs) that enable the efficient extraction and preconcentration of specific ctDNA sequences from human serum with flow rates up to 15 µL/min. We first demonstrated that implementation of a vibration system inducing flow rate fluctuations combined with a mixture of different bead sizes significantly enhanced bead homogeneity, thereby increasing capture efficiency. Taking advantage of this new generation of high-throughput magnetic FBs, we then developed a new method to selectively capture a double-stranded (dsDNA) BRAF mutated DNA sequence in complex matrices such as patient serum. Finally, as proof of concept, ligation chain reaction (LCR) assays were performed to specifically amplify a mutated BRAF sequence, allowing the detection of concentrations as low as 6 × 10^4^ copies/µL of the mutated DNA sequence in serum.

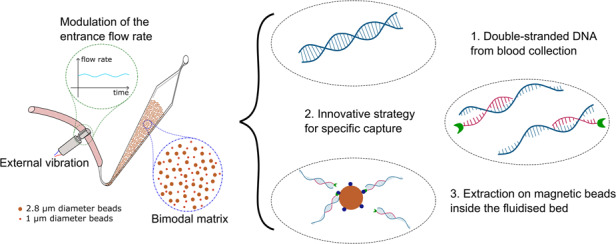

## Introduction

The detection of trace analytes such as rare biomarkers requires the development of new sample treatment modules that couple high-purity extraction to preconcentration steps and are compatible with large volume samples. Microfluidics modules have met the challenge of high sensitivity with the successful integration of diverse miniaturized technologies^1^. They also offer the possibility of reducing volumes and costs, decreasing the analysis time and optimizing the process.

Among the possible options for the microfluidic extraction of trace elements, solid phase extraction (SPE) offers an interesting solution focusing the separation on a solid support combined with high versatility and simplified compatibility with analytical devices^2^. The efficiency of the extraction method relies on an optimized interaction between the solid surface and the liquid phase, which can be increased by a structured surface^3^, porous polymer monoliths^4^ or bead packing^5^.

Among the different microfluidic approaches based on solid phase extraction, fluidized beds (FBs) present interesting features. Conventional FBs are macrosystems made of a moving fluid/gas phase in a dense array of micro or macro particles in motion^6^. Equilibrium occurs between hydrodynamic drag forces and gravitational forces that keep the particles inside the chamber. FBs are widely used in industry because they provide many benefits: continuous and homogeneous bead recirculation while avoiding system clogging, high surface contact, low backpressure and versatility in particle coating^7^. However, the microfluidic integration of FBs is not common, and only a few attempts have been reported in the literature^8–10^. Most of them consist of a downscaling of the macroscopic fluidization concept, which is poorly suitable for miniaturization because gravitational forces are negligible at the microscale. We recently proposed a new concept of a fluidized bed where magnetic forces replace gravity forces, with the solid phase hence being magnetic microbeads, resulting in a magnetic–drag force equilibrium^11^. The potential and versatility of microfluidic FBs for bioanalysis was recently demonstrated^12,13^. In particular, the first attempts to use FBs for nucleic acid analysis were reported, especially the detection of a single-stranded oligonucleotide by combining padlock probes and rolling circle amplification (RCA)^14^ and a nonspecific interaction to capture cell-free DNA (cfDNA) coupled with on-chip droplet encapsulation for ddPCR^15^. Despite those promising results, these contributions also highlight the limited throughput of the current microfluidic FBs.

Notably, the detection of a cancer biomarker, circulating tumor DNA (ctDNA), requires high sensitivity and selectivity. ctDNA, which represents a small fraction of total cfDNA, is a valuable and highly specific biomarker for disease and treatment monitoring, as it carries the somatic mutations of most cancers^16,17^. The use of ctDNA in oncology is exponentially evolving with numerous ongoing clinical trials^18^. For instance, it has been recently reported that ctDNA monitoring can predict the usefulness of adjuvant chemotherapies and guide decision-making in patient management^19–21^. The isolation and subsequent analysis of ctDNA remains a bioanalytical challenge, as ctDNA is present at low concentrations (from 5 to 0.01% of total cfDNA) within many other blood stream components^22,23^. Other body fluids contain ctDNA as well, allowing cancer monitoring using nonblood sources of ctDNA^24^. Current available methods of nucleic acid extraction involve precipitation methods, column-based techniques, magnetic beads and centrifugation^25,26^. Furthermore, to obtain information from extracted DNA sequences, these extraction methods must be combined with molecular amplification based on polymerase chain reaction (PCR) or isothermal amplification^27^. Next-generation sequencing has been increasingly used on a daily basis to further extract information from ctDNA^24,28^. These analytical methods require several pieces of equipment as well as laborious experimental steps that could each potentially lead to errors. Miniaturized platforms capable of quickly extracting ctDNA in a single-step purification from body fluids have the potential to address these challenges.

In this work, we describe the development of a new generation of FBs compatible with higher throughput to process larger sample volumes and higher flow rates. Here, we show that FB upscaling remains highly challenging as it enables new bead behaviors. New hydrodynamic settings must be considered and examined to achieve a homogeneous magnetic bead distribution within the device and consequently an efficient solid phase extraction. Two main physical approaches were investigated to maintain the FB properties in a high-throughput scaled-up device: inducing fluctuations in the system by implementing vibration at the input of the device and tuning the bead composition with a bimodal size distribution. The effect of these strategies on FB analytical performance was first assessed using a biotin–streptavidin extraction model and then on DNA-specific extraction. Finally, extraction of ctDNA from serum samples was performed and detected by the specific amplification of the target DNA by a ligase chain reaction (LCR) assay.

## Experimental section

### DNA sequences, oligonucleotides, primers and LCR probes

The DNA sequences were fragments (80 and 277 bp) of the *BRAF* gene (wild type (WT) or containing the BRAF V600E mutation (MUT)). The BRAF DNA sequence of 277 bp was used in the capture experiments from serum. The BRAF wild type (WT) and the mutated (MUT) fluorescent DNA sequences of 80 bp had an Alexa Fluor® 488 dye fixed at the 5’ end. The BRAF MUT DNA sequence (277 bp), biotinylated oligonucleotides so-called “capture probes” (20 and 80 bases), qPCR primers and LCR probes were provided by Eurofins Genomics (Ebersberg, Germany) and IDT Technologies (Leuven, Belgium). Upon reception, DNA fragments were diluted in Tris-EDTA buffer solution (pH 8.0) as suggested by the manufacturer, aliquoted and stored at −20 °C. The DNA sequences, primers and probes are shown in Table S1.

### Development of next-generation fluidized bed

The microfluidic FB, as previously reported^11^, had a height of 50 µm and a magnetic particle capacity of 50 µg. The height of the new generation of FB chips was increased from 50 to 250 µm and was filled with 250 µg of magnetic beads. More details can be found in the Supporting Information. Two approaches for bead homogenization were investigated:

#### Vibrations

**Fig. 1:**
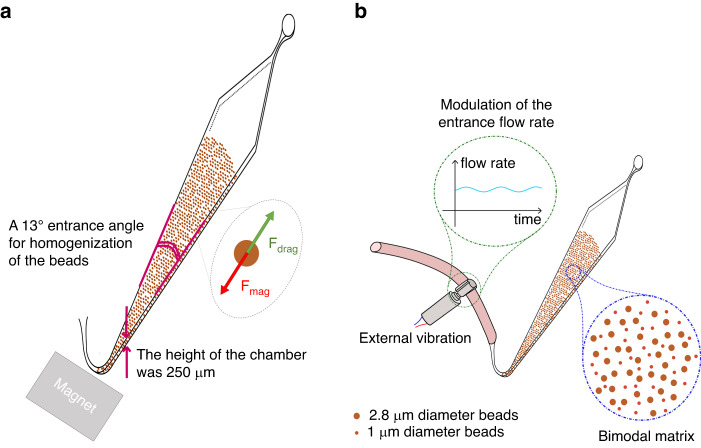
Homogenization strategies for high throughput fluidized bed. Schematic representation of **a** the fluidized bed with a representative bead in equilibrium between the drag force (F_drag_) and the magnetic force (F_mag_) and **b** the homogenization strategies (vibration and bimodal support) to increase the efficiency of the second generation of microfluidic magnetic fluidized bed

A miniature electric motor coupled to a partially off-balanced mass set to 2.4 V and 0.04 A (Model 304–101, Precision Microdrives Ltd, London, UK) was used to add vibrations. The motor was positioned on the inlet tubing as represented in Fig. 1. The vibration amplitude approached 11 g, the vibration frequency nearly reached 200 Hz, and the acceleration efficiency was 12 g/W.

#### Bimodal distribution (two sizes of beads)

Dynabeads^TM^ MyOne Carboxylic Acid (1 μm in diameter), M-450 (4.5 µm in diameter) and M-280 (2.8 μm in diameter) were used to prepare a bimodal support. Beads with different sizes were washed with T2× and T1× buffers, mixed at varying mass ratios and then injected into the FB. A detailed description can be found in the Supporting Information.

### Extraction of biomolecules using FB

#### Extraction of fluorescent biotin on streptavidin beads (streptavidin-biotin model)

A streptavidin–biotin assay was used as the first model to evaluate the effect of vibrations and bimodal support on FB-based extraction. For this purpose, Dynabeads^TM^ M-280 Streptavidin (250 µg) were washed, resuspended in the capturing buffer and then percolated inside the chips at 5 and 15 μL/min to capture the fluorescent biotin (Bt) sequence (Bt-Alexa Fluor® 488) (Table S1A). The biotin sequence was tested at concentrations ranging from 20 to 90 pmol. The dual bead composition was prepared by mixing the M-280 streptavidin beads with variable masses of Myone COOH beads (Table S2). The capture efficiency was evaluated by measuring the fluorescent signal as described in the following section.

#### Extraction of a fluorescent ssDNA sequence of the BRAF gene

A second series of experiments was conducted to demonstrate the potential of this new FB. Briefly, single-stranded DNA was specifically extracted in FBs by hybridization on beads grafted with complementary oligonucleotides. First, the effects of the hybridization temperature (49 and 59 °C), length of the biotinylated oligonucleotide (20 to 80 bases) and NaCl concentration (100 nM to 1 M) in the Tris-HCL buffer were studied to optimize the capture conditions. For extraction, Dynabeads^TM^ MyOne Streptavidin T1 (250 µg) were functionalized with a biotinylated sequence of 80 bases and then percolated inside the chip. Control experiments were also performed by using nonfunctionalized beads. The temperature was set to 49 °C. A WT or MUT fluorescent DNA sequence of 80 bases of the BRAF gene (Table S1B and C) was prepared at a concentration of 10 nM in TRIS-HCL buffer containing 1 M NaCl and injected into the FB at 5 and 15 μL/min to measure the fluorescence intensity. This fluorescence intensity was considered to be that of noncaptured DNA on beads and was compared to an initial DNA solution previously injected into the chip at the same flow rate for approximately 5 min. The capture efficiency was determined as follows:

1 $${\rm{Capture}}\; {\rm{efficiency}}( \% )=\frac{\left({I}_{0}-{I}_{{nc}}\right)}{{I}_{0}}\times 100$$

where *I*_0_ is the fluorescence intensity of the initial DNA solution (calibration measurement without any bead in the system) and *I*_*nc*_ is the fluorescence intensity of the solution of noncaptured DNA. All experiments were performed on the microfluidic platform installed on the microscope and repeated at least 3 times. More details on the DNA extraction are provided in the Supporting Information.

#### Capture of ctDNA from human serum

The procedure for ctDNA extraction from human serum is shown in Fig. 6. Prior to step 1, serum samples were incubated with proteinase K (2.75 mg/mL) for 2 h at 37 °C to digest the serum proteins. During step 1, a mutated nonfluorescent dsDNA 277 bp and biotinylated capture probes at 0.4 µM were added to the serum (Table S1H, I). The tests were performed at DNA concentrations of 100 fM (6 × 10^4^ copies/µL) and 1 pM (6 × 10^5^ copies/µL). After 5 min of heating at 95 °C and incubation at room temperature (RT) for 15 min (steps 3 and 4), the spiked serum was perfused through the expended FB (150 µl at 5 µL/min) containing MyOne Streptavidin beads (250 µg). For further analysis (step 5), a tube connected to the output of the chip allowed us to collect the serum after the capturing step. The beads were rinsed with PBS buffer at 5 µL/min for 20 min and then pushed out of the chip and collected to perform amplification through the LCR method. Serum samples were analyzed by SYBR-green PCR quantitative assay. The experiments were repeated 3 times and carried out on the FB automated platform (see details in Supporting Information for description of the platform on Fig. S1 and complete ctDNA capture from serum).

### Analyses

#### Quantitative polymerase chain reaction (qPCR) assay

All qPCR experiments were carried out on a Cepheid^TM^ SmartCycler^TM^ Real-Time PCR detection system (Cepheid Inc., Sunnyvale, CA). The amplification method used for thermocycling was as inspired by^29^ an initial denaturation step of 5 min at 95 °C, followed by 40 cycles of 95 °C for 15 s and 57 °C for 60 s. Each reaction contained the following reagents: 10 µL of KiCqStart SYBR^TM^ Green ready mix (2×), 3.0 µL of UltraPure^TM^ DNase/RNase-free distilled water, 1.0 µL of 10 µM forwards and reverse primers (Table S1J), and 5.0 µL of template DNA.

#### Ligase chain procedure (LCR)

##### Sample preparation before LCR and target release from beads

Beads were mixed with 50 µL of 4 µM biotin diluted in PBS and incubated for 15 min at room temperature. The sample with beads was placed in a magnetic rack and washed up to 3 times with 50 µL of PBS by removing the supernatant each time. During the last washing step, the beads were resuspended in 16 µL of ultrapure water. The bead sample was then heated at 95 °C for 3–5 min to release the captured target. Then, heating was followed by immediate transfer of the sample in a magnetic stand placed into ice to prevent the reassociation of the target with the capture probes, and 15 µL of supernatant was removed and subjected to LCR.

##### LCR assay

Template (15 µL of supernatant with the released target, 2.5 µL of the initial template in human serum, 2.5 µL of the output in human serum or no template) was mixed with LCR mix consisting of 1× AmpLigase Thermostable DNA Ligase buffer, 1× BSA, 80 nM of each of the 4 probes (Table S1K to N) and 1.25 units AmpLigase Thermostable DNA Ligase. Three of the four probes were 50 nt each in length (p1, cp1, cp2). The fourth probe (p2) was 68 nt in length, with a 3′ end ssDNA tail of 18 nt. The single strand DNA tail was added to enhance the specificity of LCR. The first pair of probes p1 and p2 was complementary to the forwards strand of BRAF, and the second pair cp1 and cp2 was complementary to the reverse strand, while the two pairs were also complementary to each other. Upon hybridization of the 4 probes with the dsDNA 277 target, the probes were ligated, giving rise to a product of 100 bp with a 3′ ssDNA tail of 18 nt. LCR reactions had a total volume of 25 µL and were subjected to 30 cycles of denaturation at 92 °C for 5 s followed by annealing and ligation at 65 °C for 5 s. LCR products were analyzed by gel electrophoresis.

### Data analysis

All experiments were performed at least in triplicate. The analyses were performed with GraphPad Prism 9.5.1 software (GraphPad Software, Boston, USA). Comparisons were performed with the two-way ANOVA function of the software. Data are presented as the mean ± SD. The statistical significance was determined with two-way ANOVA. The statistical significance was determined with a t test as indicated: ****p < 0.0001, ***p < 0.001, **p < 0.01, *p < 0.05.

## Results and discussion

### Microfluidic fluidized bed for high throughput analysis

The existing microfluidic fluidized bed technologies have specific features, such as low backpressure, continuous bead recirculation, and high bead density, that make them potential technologies for ctDNA extraction^10,14,15^. As illustrated in Fig. 1, the magnetic beads achieve an equilibrium state between the drag force resulting from fluid percolation and the magnetic force exerted by a permanent magnet placed at the entrance of the chip. This dynamic equilibrium enables the movement of the beads within the microfluidic chamber, which imparts distinctive properties such as low backpressure, bead recirculation, and tuneable bead density.

However, thus far, their analytical throughput is highly limited by the applicable range of flow rates. Pereiro et al. showed that for flow rates higher than 3 µL/min, the drag force overcomes the magnetic force, and a fraction of the magnetic beads could be taken away^11^. At 5 µL/min, all the beads were dragged away. This flow rate limitation is a major drawback when considering large-volume samples (typically above 50 µL). Here, we report on the next generation of microfluidic FBs exhibiting higher working flow rates and increased specific surface, making them compatible for trace analysis. To scale-up the device, different strategies were considered, aiming specifically to increase the volume of the microfluidic chamber. Previous work demonstrated that the chamber geometry is critical: a specific angle of 13° at the entrance of the chip was found to be preferable to obtain a homogenous distribution of the magnetic field lines and maintain particle mobility inside the chamber^11^. To maintain the balance between the magnetic field and the hydrodynamics, we thus designed a new generation of microfluidic FBs by keeping all the 2D dimensions constant while only increasing the chamber height. A modification of the height of the chamber allows the flow rate to be increased without changing the fluid velocity and thus without changing the balance between the drag and magnetic force (the latter remaining unchanged). The height was increased fivefold, from 50 to 250 µm (Fig. 1a). This upscaling was expected to allow higher flow rates while increasing the bead quantity and consequently the total specific surface of the solid phase.

To validate our initial assumption, we first characterized the bead distribution and dynamics within the FB for the two chip heights (50 and 250 µm). We observed bead recirculation within the microchamber independently of the chip height. The recirculation relies on the dynamic equilibrium between forces inside a diamond geometry, as previously described^11^. At 1 µL/min, the 50 µm height FB presents a homogenous distribution of the magnetic beads within the chamber (Fig. S2A). However, preferential pathways with less concentrated bead regions were observed in the 250 µm height FB (Fig. S2, C), and this heterogeneous distribution was amplified at higher flow rates (above 5 µL/min). This heterogeneity of the bead distribution within the chamber could be related to the Poiseuille flow profile with an increased dispersion of the flow velocity distribution along the z-axis compared to 50 µm chips.

The presence of such fractures within the bed of beads reflects areas of lower local hydrodynamic resistance and is expected to be deleterious for SPE, as it will prevent homogeneous percolation of the sample through the whole bed. These observations were quantitatively confirmed by an on-chip bead-based streptavidin–biotin extraction assay. Our results showed that at 1 µL/min, the biotin capture rate drops from 90% with the 50 µm high FB (at 5 µL/min) to 60% for the 250 µm FB. To restore a homogenous distribution of magnetic beads in the upscaled FB chamber, mixing strategies, either passive or active, have been investigated.

### Improving bead homogeneity within the FB by modulation of the flow

One of the simplest ways to induce mixing in microdevices is to induce a modulation of the liquid pressurization by different means, such as the addition of bubbles^30^, segmented flow^31,32^, or internal micropumps^33^, usually linked to complex microfabrication and issues with control of temperature inside the channels. We first studied how a modulation of the inlet pressure would affect bead homogeneity. As the injection is controlled by pressurized inlets, the modulation was performed through direct modification of the applied pressure thanks to a feedback-loop system achieved with a flowmeter placed at the chip outlet; thus, we were able to continuously open and close the FB. Our results showed that, under these conditions, an increase in the frequency of the pressure modulation improved the capture efficiency of the fluidized bed. This suggests that increasing the mobility of beads and modifying the flow pattern in the bed helps improve the solid/liquid exchange. However, in our FB system, directly modulating the inlet pressure can induce a burst of pressure to break the interactions between the beads and resuspend them in the liquid. This can lead to a sudden expansion of the FB and induce the loss of beads.

To reach higher frequencies of flow modulation while improving the experimental reproducibility, a vibration system was implemented on the inlet tubing (Fig. 1b). We expected this system to induce reproducible mechanical stimulation on the elastic tubing due to the repetitive strike of the decentered mass of the vibration system resulting in tube deformation and consequently periodic fluctuations of the flow rate. The vibrating system (vibration amplitude of 11 g, frequency of 200 Hz) used here is similar to those found in smartphones. Its low-cost simple implementation is advantageous in the context of analytical applications such as the one targeted in this study.

**Fig. 2:**
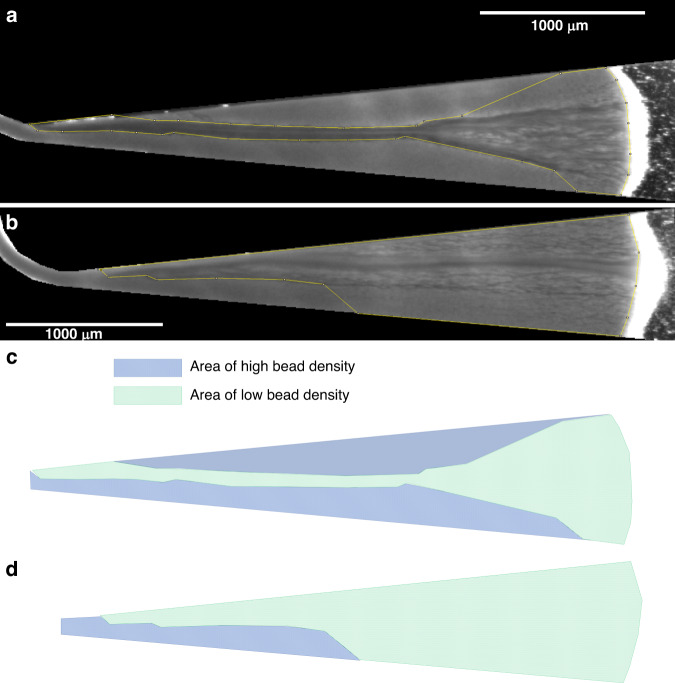
Impact of the flow modulation on the percolation area in the fluidized bed. Picture of a 250 µm chip with 250 µg of streptavidin M-280 beads at 5 µL/min **a** without vibration and **b** with vibration, where the areas of low bead density are circled in yellow and their planar projection **c** without and **d** with vibration

**Fig. 3:**
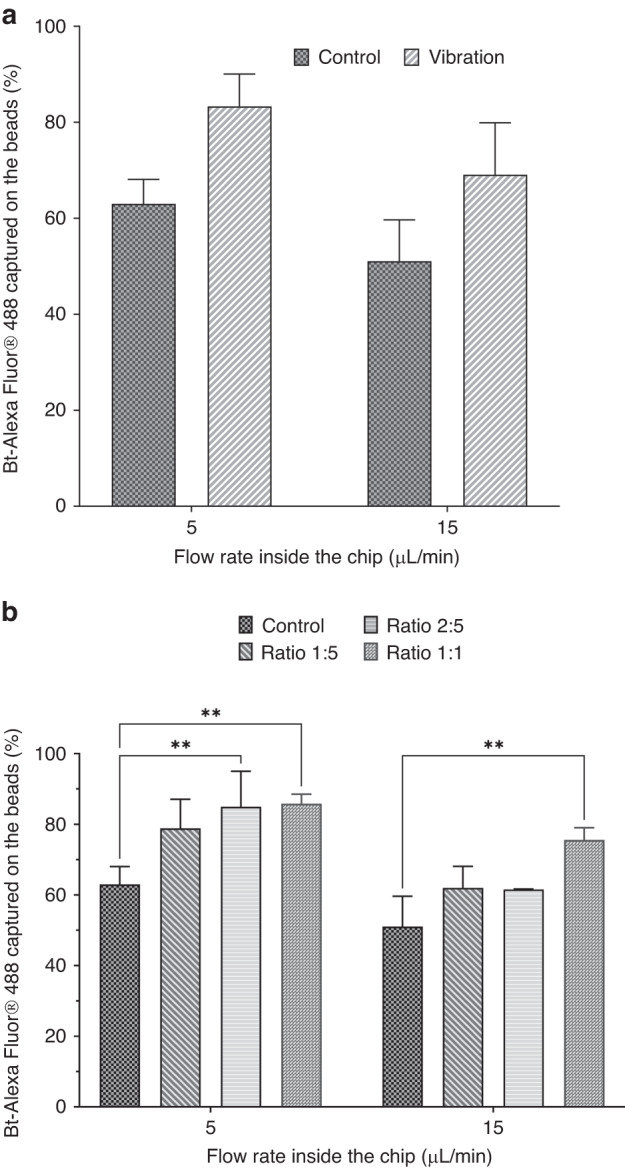
Influence of the homogenization strategies on the capture of biomolecules in the fluidized bed. **a** Vibration Modality. Variation in the capture efficiency after flowing 20 pmol of Bt-AlexaFluor488® inside the matrix of streptavidin beads at 5 and 15 µL/min with and without vibration. **b** Dual bead composition, with indication in the legend of the 1 µm: 2.8 µm bead ratio. Variation in the capture efficiency after flowing 20 pmol of Bt-AlexaFluor488® as a function of bead composition (*p* < 0.01). The ratio is relative to the quantity of MyOne COOH beads added to the control system (as described in Table S2)

In the presence of vibrations on the inlet tubing, an increase in the surface percolated by the fluid is observed (Fig. 2). At 5 µL/min, the area of low bead density was more homogenously distributed along the axis of the flow with a 2.5-fold increase in the projected surface (Fig. 2d) compared with conditions in the absence of vibration (Fig. 2c). This confirms that by tuning the frequency and amplitude of the oscillations, we can induce a rapid modulation of the flow rate within the fluidized bed and consequently disturb the fluidization process, with a combination of shear stress and the expansion/compaction mechanism of the bed. Similar to previous experiments, a streptavidin–biotin extraction assay was performed to evaluate the effect of vibrations on FB-based extraction. A series of experiments were performed by injecting fluorescent biotin (20 pmol) into the upscaled FB at flow rates of 5 and 15 µL/min in the presence and absence of vibrations. Our results suggest that inducing flow fluctuations through the presence of vibration seems to improve the capture efficiency by approximately 20%, even at a flow rate as high as 15 µL/min (*p* = 0.064, Fig. 3a).

### Improving the bead homogeneity by bimodal bead size

Despite the significant improvement offered by the implementation of flow fluctuations with the vibrating system, the ability of the FB to efficiently capture biomolecules remained limited, especially when considering further ctDNA application. Passive strategies to improve mixing within the FB were thus investigated. It has already been shown that the particle size distribution could play a major role in the fluidized bed properties. A commonly observed phenomenon in conventional macroscopic fluidized beds is the spatial segregation of the beads as a function of their size^34^. With specific parameters, working with different sizes of particles has also been shown to improve the mixing within macroscopic gravity-based fluidized beds^35^.

We thus investigated how the use of a mixture of beads with various sizes could affect bead homogeneity within the microfluidic FB. Different sizes of beads (1, 2.8, and 4.5 µm in diameter) with different magnetic susceptibilities (Table S3) were mixed at varying mass ratios (1:1, 1:5 and 2:5, w/w) while keeping the mass of the functionalized beads constant to always have the same available surface for capture. First, we noticed that the beads with the largest diameter (4.5 µm) were not distributed homogenously when mixed with smaller beads. As illustrated in Fig. S3, smaller beads (2.8 µm) are found close to the entrance of the fluidized bed, while most of the 4.5 µm beads are observed at the edge of the bed of beads. This segregation appeared for any mixture of 4.5 µm beads, whereas such an effect was not observed for bead suspensions made of 1 µm and 2.8 µm beads (Fig. S4). This is related to a modification of the equilibrium of forces at the scale of individual beads. In contrast, our data showed that increasing the quantity of 1 µm beads (from a 1:5 to 1:1 ratio, w/w) in a dispersion of 2.8 µm beads had a significant effect on the bead distribution within the FB. An increased percentage of 1 µm beads leads to a more homogeneous bead distribution and to an increased bed projected area even at 15 µL/min (Fig. S4). This observation is consistent with the model developed by Petousis et al.^36^. This model aims to describe the dynamics of magnetic bead assembly, including their rotation and ruptures, based on a bimodal bead size distribution. They showed that columns made of beads of different sizes or magnetic susceptibilities provide more fragile bundles of particles upon shear stress and thus promote particle mobility in the bed.

In the microfluidic FB, the beads used are superparamagnetic, and they self-assemble as columns under a magnetic field due to dipole‒dipole interactions^32^. Magnetic beads of different sizes and susceptibilities could organize either as two ‘populations’ of columns made of large or small beads exclusively or more likely as columns made of a mix of the different types of beads. Both situations are favorable for bed homogeneity: either the smaller beads fill the void between the larger beads or they insert themselves inside the assembly of larger beads. In both cases, they are expected to change and perturb the local hydrodynamic resistance, which should improve the bed homogeneity.

To quantify the effect of using a bimodal bead size composition, we investigated the effect of bead composition on the capture efficiency with the streptavidin–biotin model assay (Table S2). Those experiments were performed with 250 µg of 2.8 µm streptavidin beads and a given amount of 1 µm carboxylic acid beads that did not interact with biotin (data not shown). Our results showed that the use of a bimodal bead composition significantly improved (*p* < 0.01) the FB capture efficiency compared to the control at ratios of 2:5 and 1:1 (Fig. 3b). At 5 µL/min, the capture efficiency increased with the percentage of 1 µm beads from 66 to 85% for 1:5 and 1:1 (w/w) 1 µm beads, respectively. At 15 µL/min, the use of a bimodal bead mixture increased the capture efficiency from 52% (monomodal beads) to 75% (bimodal, ratio 1:1, w/w) (Fig. 3b).

### Combining passive and active strategies to improve FB performance

**Fig. 4:**
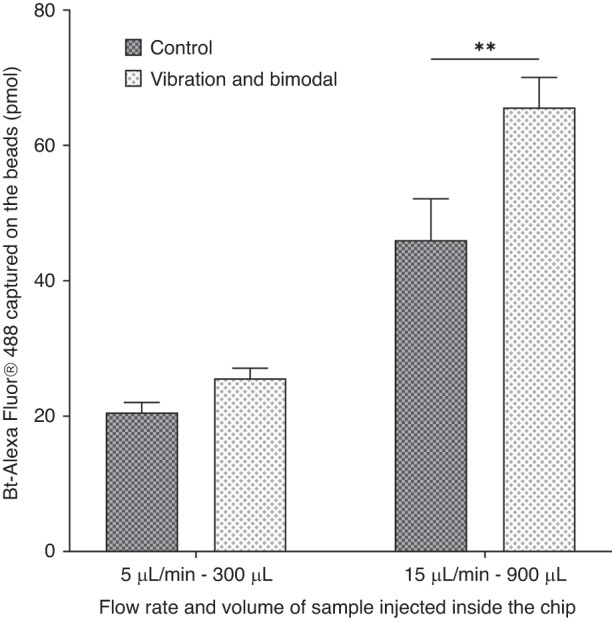
Influence of the combined homogenization strategies on the capture of biomolecules. Influence of the combination of vibration and bead composition on the quantity of Bt-AlexaFluor488® captured at different flow rates for an experiment limited in time (1 h). The control refers to an experiment without vibration and only one bead size (M-280)

Both these active and passive approaches demonstrated their potential to improve the bead distribution within the FB and consequently its ability to efficiently capture biomolecules flowing through the FB. As both strategies rely on different mechanisms, we assumed that a combination of both could even further improve FB performance. Our assumption was assessed, as previously described, with a streptavidin–biotin model assay (Fig. 4).

As shown in Fig. 4, the combination of the bimodal and vibration strategies drastically improved the biotin capture efficiency with a striking and significant (*p* < 0.01) improvement at 15 µL/min. To account for the assay throughput, all of these experiments were performed for one hour. At 15 µL/min, 90 pmol of biotin was injected into the microfluidic FB. When working either with vibrations or bimodal support, 53 pmol of biotin was extracted, matching a capture efficiency of 59% (data not shown). However, with the combination of them, a capture efficiency of 73% is achieved with more than 65 pmol captured. At 5 µL/min, for a one-hour extraction experiment, 30 pmol of biotin was injected into the chip; 25.6 pmol was extracted with the addition of both vibration and a bimodal matrix (capture rate ~85%) against 20.5 pmol in the control. These results suggest that regardless of the flow rate, the combination of flow fluctuations and bimodal size support drastically improves the extraction performance with the FB. However, it is also worth mentioning that the capture efficiency remains higher when working at 5 µL/min. This is of course expected given that the residence time of an analyte is directly proportional to the sample flow rate. However, all in one, for one hour of experiment despite lower capture efficiency, the quantity of analyte extracted is higher when working at 15 µL/min. Depending on the nature of the sample and the downstream analysis to be performed after the extraction, the FB can be used either at low or high flow rates.

**Fig. 5:**
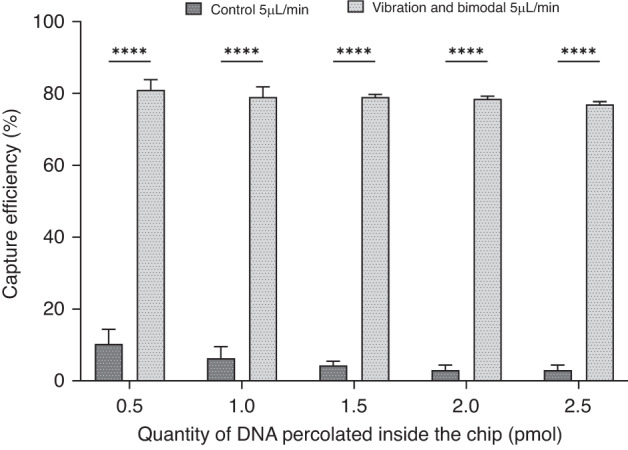
Influence of the combined strategies on the DNA capture efficiency by hybridization. DNA capture efficiencies by hybridization on beads at 49 °C as a function of the quantity of DNA percolated inside the FB (250 µm height) at 15 µl/min. The vibration and bimodal method is compared to the control. DNA was a fluorescent WT sequence (80 bases) of the BRAF gene (*p* < 0.0001)

To move beyond the model streptavidin/biotin assay and to further demonstrate the potential of this approach for bioanalysis, a series of experiments was conducted to extract single strand DNA by specific hybridization with beads grafted with complementary oligonucleotides. The experiments were performed under optimized capture conditions (Table S4, Fig. S5) at 15 µL/min. The effect of the homogenization approaches on the capture efficiencies of specific ssDNA performed at 49 °C is shown in Fig. 5. The target is the fluorescent DNA sequence (80 bases) of the BRAF gene. Similar to streptavidin–biotin extraction, the capture efficiency is drastically improved (*p* < 0.001) when combining a vibration system and a bimodal support compared to the control conditions, as shown in Fig. 5. This combination allowed capture efficiencies of 81% to be reached compared to those of 35% obtained with the control at a flow rate of 15 µL/min, and similar experiments was performed at 5 µL/min (Fig. S6). These results confirmed the potential of our microfluidic approach to specifically extract DNA at high throughput.

### Selective capture of dsDNA *BRAF* target in human serum using FB followed by LCR amplification

**Fig. 6:**
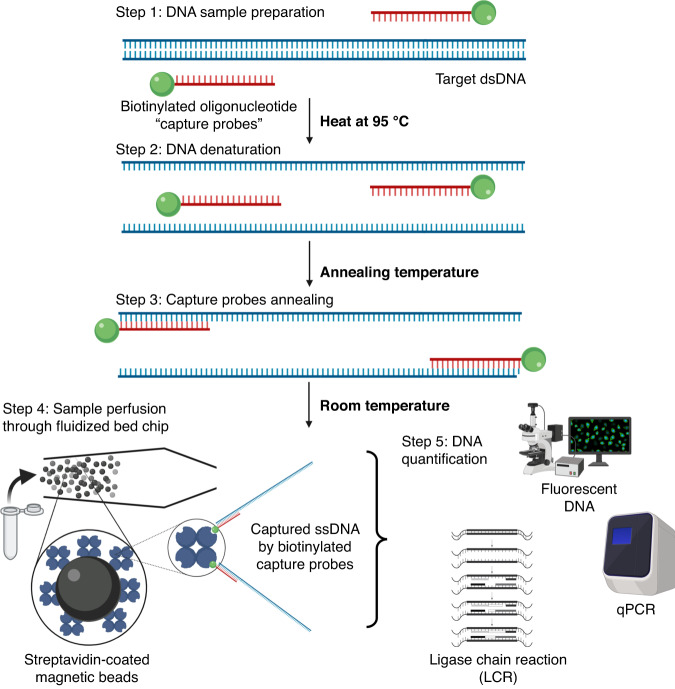
Double-stranded ctDNA capture strategy in biological samples. Schematics of the double-stranded ctDNA capture strategy in biological samples. Step 1: The sample containing the target DNA and biotinylated capture probes are mixed. Step 2: Heating at 95 °C denatures the double-stranded DNA. Step 3: Decrease the temperature until the capture probe annealing temperature to specifically hybridize each target ssDNA to the biotinylated capture probes. Step 4: After capture probes are annealed to the target DNA, the sample is injected into the device, which is filled with streptavidin-coated magnetic beads, at room temperature. High streptavidin–biotin affinity will result in target DNA capture onto the beads. Step 5: DNA detection and quantification is done either by fluorescence measurements, qPCR, or LCR assays (pictures created using BioRender application)

We next designed a new strategy compatible with patient sample analysis. It aims to extract specific sequences of double-stranded DNA (dsDNA) from simple or complex samples such as human serum. Our approach relies first on the denaturation of dsDNA followed by the hybridization of each single strand with biotinylated complementary capture probes specific to the targeted sequence; this first step is performed off-chip (Fig. 6). More precisely, after mixing the sample with specific capture biotinylated probes (step 1), a denaturation step (95 °C for 5 min) is performed (step 2). The sample is then cooled down so that each capture probe will specifically hybridize to its complementary sequence on each ssDNA with the formation of biotinylated capture probe–DNA complexes. Those complexes are next injected into the microfluidic FB that contains streptavidin-coated magnetic beads for extraction (step 4). Finally, we demonstrate the compatibility of the specific dsDNA FB extraction with downstream amplification and detection using qPCR or ligase chain reaction (LCR).

**Fig. 7:**
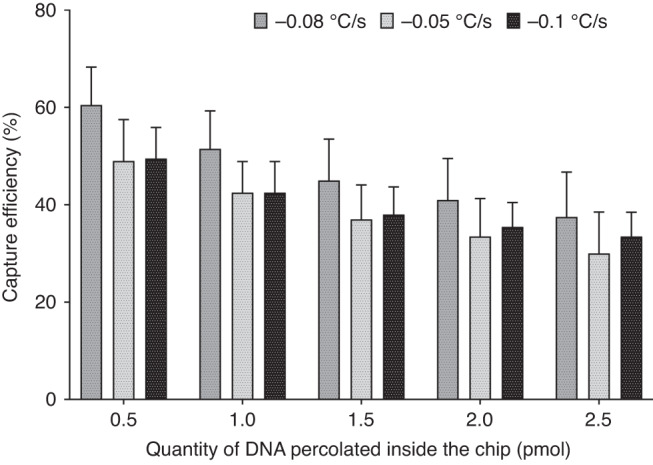
Influence of the cooling rate on the capture efficiency of a double-stranded ctDNA sequence in the fluidized bed. Capture efficiencies of the fluorescently mutant dsDNA sequence of 80 bp of the BRAF gene after hybridization at different cooling rates: −0.1, −0.05, and −0,08 °C/s up RT. ctDNA was prepared at 10 nM in 10 mM Tris-HCL buffer. Capture was performed in an FB chip at 15 µL/min with vibration and bimodal support (*p* > 0.05)

In this approach, the cooling step is critical for the specific capture probes to anneal with the target. Indeed, after denaturation of target dsDNA, DNA can either anneal to the capture probes or rehybridize itself. The cooling conditions were therefore optimized as well as the initial capture probe design and concentration. For this purpose, a double-stranded fluorescent *BRAF* V600E sequence of 80 bp (Table S1C) prepared at 10 nM in Tris-HCL buffer was used as a model target and captured according to the procedure described in Fig. 6. After denaturation at 95 °C, samples were cooled to room temperature (25 °C) for hybridization using different cooling rates: −0.1 °C/s, −0.05 °C/s or −0.08 °C/s. After hybridization, the target DNA-capture probe hybrids were immediately injected into the FB chip at 15 µL/min to proceed to the extraction on streptavidin beads. Our results showed that for an initial target of dsDNA of 0.5 pmol (25.2 ng), a capture efficiency of 60.5 ± 7.8% was achieved when the cooling rate was −0.08 °C/s, while 49.5 ± 6.4% and 49.0 ± 8.5% (*n* = 3) capture efficiencies were obtained with cooling rates of −0.01 °C/s and −0.05 °C/s, respectively (Fig. 7). Thus, we decided to carry out dsDNA extraction from serum samples with a cooling rate of 0.08 °C/s for 15 min.

To process serum samples, proteinase K sample pretreatment was optimized to avoid precipitation of the protein serum content during the denaturation step (Fig. S7). Pretreatment with proteinase K (2 h at 37 °C) was sufficient to digest the serum proteins, allowing sample analysis without requiring any dilution step.

To assess the performance of this sample preparation combined with our next generation of fluidized bed, we first used an indirect approach by qPCR to quantitatively measure the dsDNA capture efficiency (mat & met). The average BRAF V600E extraction efficiency was estimated to be 44% ± 0.1% and 46 ± 1.5% for initial concentrations of 100 fM (17 pg/mL) and 1 pM (171 pg/mL) spiked in human serum, respectively. As the serum is a complex matrix with many other biomolecules (proteins, RNA, cfDNA), we hypothesize that a screening effect may affect the extraction rate; however, it is worth mentioning that the extraction efficiency remains quite high compared to existing technologies^37^.

**Fig. 8:**
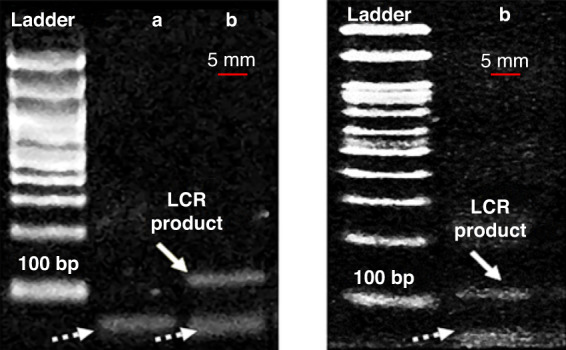
Detection of LCR products by gel electrophoresis after capture of dsDNA BRAF target in serum samples in the fluidized bed. Detection of 1 pM (6 × 10^5^ copies/µL) (left) and 100 fM (6 × 10^4^ copies/µL) (right) dsDNA 277 bp BRAF V600E target by gel electrophoresis (2% agarose gel) of LCR products (white arrows) after capture in serum samples by fluidized bed. LCR probes are represented by white dotted arrows. Left -> Right: Ladder, LCR on-beads **a** LCR of heat-released target **b**. The expected product size is 100 bp

Following this first evaluation of the capture efficiency of ds DNA spiked in serum samples, we next demonstrate how the captured DNA can be released from the beads and detected after undergoing enrichment by the LCR step. LCR is a highly specific DNA amplification method known for its ability to amplify point mutations and single nucleotide polymorphisms and can serve as an alternative to DNA polymerase-based amplification^38–40^. For LCR, a DNA ligase and two pairs of probes that are fully complementary to the mutant DNA target but not to the WT sequence were used. The pair of probes p1 (50 bases) and p2 (68 bases) were complementary to the forwards strand of the mutant BRAF sequence, and the other probes (cp1 and cp2, both 50 bases) were complementary to the reverse strand. Upon hybridization of the probes with the target, the ligase connects the two adjacent oligos only when perfect complementarity occurs (Fig. S8). Multiple cycles of denaturation, annealing and ligation result in exponential amplification of the target DNA carrying the point mutation. Two strategies were considered: (i) LCR performed directly on beads or (ii) performed after heat release of the captured target from the beads. LCR products were visualized by gel electrophoresis only after the heat release of the DNA from beads, as shown in Fig. 8. We detected a band at the expected molecular weight (100 bp) for 1 pM (or 6 × 10^5^ copies/µL) and 100 fM (or 6 × 10^4^ copies/µL) *BRAF* V600E target, demonstrating the ability of our approach to capture/release and amplify a specific sequence in human serum.

## Conclusion

Here, we developed a new generation of FBs with larger chambers to accommodate higher flow rates than those used for the first generation of FBs and consequently larger sample volumes. Due to its fluidization regime, the new FB provides continuous and homogenous recirculation of magnetic beads, avoiding bead aggregation and system clogging. We demonstrated that the use of a magnetic bimodal support coupled with flow fluctuations induced by a vibrating system improved the capture efficiency with a flow rate up to 15 µL/min, as observed both with a biotin–streptavidin model and with the capture of a ssDNA target by hybridization on beads. As a proof of concept, we then extracted clinically relevant concentrations of *BRAF* V600E from biological samples with a new ctDNA extraction strategy enabling the extraction of specific sequences of dsDNA. Specifically, we isolated *BRAF* V600E sequences in low concentrations directly from serum samples and demonstrated the compatibility of our system with the specific amplification of the DNA target by LCR assay. This allows the detection of a dsDNA mutant sequence at a concentration of 100 fM (6 × 10^4^ copies/µL) in human serum. The results demonstrated that the captured DNA can be released for further amplification and detection with a highly specific amplification method.

Altogether, we demonstrated how physical modification of the bed of particles and of the flow pattern can be exploited to develop a device able to specifically capture and detect dsDNA in human serum, applied here to the *BRAF* gene but transferrable to any other DNA sequence. Our system combines the versatility and simplicity of using magnetic beads for the specific extraction of DNA strands for selected double-stranded targets at the microscale. The efficiency of extraction is comparable to that in published works, with clinically relevant detection limits^41,42^. Only a few publications focus on ctDNA extraction, requiring either larger volume samples^43^ or longer preprocessing^44^. We believe that this work fills a gap between the usual microfluidics extraction platform and the clinical need to specifically work with ctDNA, opening new possibilities for cancer monitoring in routine clinical practice.

### Supplementary information

Supporting Information
