# Supplementary material for: High-throughput extraction on a dynamic solid phase for low-abundance biomarker isolation from biological samples
Source: Microsyst Nanoeng. 2023 Sep 6;9:109. doi: 10.1038/s41378-023-00582-4 (PMC10480215; doi:10.1038/s41378-023-00582-4)
Supplement: Supplementary file 1 — Supporting Information [file 41378_2023_582_MOESM1_ESM.docx]

**Supporting Information**

**High-throughput extraction on dynamic solid phase for low-abundance biomarker isolation from biological samples**

Lucile Alexandre^a,b^‡, Monica Araya-Farias^a,b^‡, Manh-Louis Nguyen^a,b^, Nikoletta Naoumi^c,d^, Giacomo Gropplero^a,b^, Electra Gizeli^c,d^, Laurent Malaquin^e^, Stéphanie Descroix ^a,b^*

^a^ Laboratoire Physico-Chimie Curie, CNRS UMR 168, Institut Curie, PSL Research University, Paris, France,
^b^ Institut Pierre-Gilles de Gennes for Microfluidic (IPGG), Paris, France,
^c^ Dept. of Biology, University of Crete, Heraklion, Greece,
^d^ Institute of Molecular Biology and Biotechnology (IMBB) & FORTH, Heraklion, Greece,
^e^ Laboratoire d’analyse et d’architecture des systèmes (LAAS) CNRS, Elia Group, Toulouse, France

**Table of content**

Table S1………………………………………………………………………………….S2

Table S2………………………………………………………………………………….S3

Table S3………………………………………………………………………………….S4

Table S4………………………………………………………………………………….S4

Figure S1………………………………………………………………………………....S5

Figure S2…………………………………………………………………………………S5

Figure S3…………………………………………………………………………………S6

Figure S4…………………………………………………………………………………S7

Figure S5………………………………………………………………………………....S8

Figure S6………………………………………………………………………………....S9

Figure S7………………………………………………………………………………....S10

Figure S7………………………………………………………………………………....S11

Chemicals and materials………………………………………………………………....S12

Washing and capturing buffers………………………….…………………………….....S12

Equipment.…………………….……………………….….……………………………..S12

Microfluidic FB platforms……………………………………………………………….S13

Microfluidic FB operating process: system preparation…………………………………S13

Optimization of capture conditions……………………………………………………....S14

Fabrication of microchips…….……….…………………………………………………S14

Serum samples………….…….……….………………………………………………....S14

**Table S1.** DNA sequences, biotinylated oligonucleotides, qPCR primers and LCR probes.

| **Sequence name** | | **Sequence (5’ to 3’)** |
| --- | --- | --- |
| **A** | Bt-AlexaFluor488 | Biotin-CTCTCTCTCTCTCTCTCTCT-AlexaFluor488 |
| **B** | BRAF-WT-80b  5’ AlexaFluor488 | TTCATGAAGACCTCACAGTAAAAATAGGTGATTTTGGTCTAGCTACAG**T**GAAATCTCGATGGAGTGGGTCCCATCAGTTT |
| **C** | BRAF-MUT-80bp  5’ AlexaFluor488 | TTCATGAAGACCTCACAGTAAAAATAGGTGATTTTGGTCTAGCTACAG**A**GAAATCTCGATGGAGTGGGTCCCATCAGTTT |
| **D** | BRAF-MUT-277 | ACCTAAACTCTTCATAATGCTTGCTCTGATAGGAAAATGAGATCTACTGTTTTCCTTTACTTACTACACCTCAGATATATTTCTTCATGAAGACCTCACAGTAAAAATAGGTGATTTTGGTCTAGCTACAG**A**GAAATCTCGATGGAGTGGGTCCCATCAGTTTGAACAGTTGTCTGGATCCATTTTGTGGATGGTAAGAATTGAGGCTATTTTTCCACTGATTAAATTTTTGGCCCTGAGATGCTGCTGAGTTACTAGAAAGTCATTGAAGGTCTCA |
| **E** | **Biotinylated oligonucleotides**  WT-20  5’ Biotin | CCCACTCCATCGAGATTTC**T** |
| **F** | WT-80  3’ Biotin | AAGTACTTCTGGAGTGTCATTTTTATCCACTAAAACCAGATCGATGTC**A**CTTTAGAGCTACCTCACCCAGGGTAGTCAAACTTCTC |
| **G** | MUT-80  3’ Biotin | AAACTGATGGGACCCACTCCATCGAGATTTC**T**CTGTAGCTAGACCAAAATCACCTATTTTTACTGTGAGGTCTTCATGAA |
| **H** | MUT-fw-80  5’ Biotin | ATGAAGACCTCACAGTAAAAATAGGTGATTTTGGTCTAGCTACAG**A**GAAATCTCGATGGAGTGGGTCCCATCAGTTTGAA |
| **I** | MUT-rv-80  5’ Biotin | TTCAAACTGATGGGACCCACTCCATCGAGATTTC**T**CTGTAGCTAGACCAAAATCACCTATTTTTACTGTGAGGTCTTCAT |
| **J** | **qPCR primers**  Forward  Reverse | ACCTAAACTCTTCATAATGCTTGC  TGAGACCTTCAATGACTTTCTAG |
| **K** | **LCR probes**  p1 | GGATCCAGACAACTGTTCAAACTGATGGGACCCACTCCATCG AGATTTCT |
| **L** | p2 | CTGTAGCTAGACCAAAATCACCTATTTTTACTGTGAGGT CTTCATGAAGAGGG GGTAGGAGTGTCGTT |
| **M** | cp1 | GAAATCTC GATGGAGTG GGTCCCATCAGT TTGAAC AGTTGTCTG GATCC |
| **N** | cp2 | TCTTCATGAAGACCTCACAGTAAAAATAGGTGATTTTGG TCTAGCTACAGA |

^a^Nucleotide T is the wild type sequence whereas A is the mutated one

^b^WT refers to the wild-type sequence of BRAF gene containing the base T (framed above)

^c^MUT refers to the mutated sequence of BRAF gene, where the normally T is switched to a A (framed above)

Table S2. Composition of the bimodal matrix of beads for the effect of a bimodal support inside the fluidized bed. The mass of M-280 Streptavidin-coated is kept constant in the 2^nd^ generation of fluidized bed chips.

| **Name** | **Mass of Dynabeads^TM^ M-280 Streptavidin (μg)** | **Mass of Dynabeads^TM^ MyOne COOH (μg)** |
| --- | --- | --- |
| Control | 250 | 0 |
| Ratio 1:5 | 250 | 50 |
| Ratio 2:5 | 250 | 100 |
| Ratio 1:1 | 250 | 250 |

Table S3. Characteristics^a^ of the magnetic beads^1^ used for the bimodal support including their effective radius, their magnetic susceptibility and their $\boldsymbol{\Delta}\boldsymbol{\chi}\boldsymbol{R}^{\boldsymbol{2}}$.

| **DynaBeads^TM^** | **Radius**  **(μm)** | **Magnetic susceptibility** $\boldsymbol{\Delta\chi}$***10^-5^ (m^3^/kg)** | $\boldsymbol{\Delta\chi}\boldsymbol{R}^{\boldsymbol{2}}$***10^-23^**  **(m^6^/kg)** |
| --- | --- | --- | --- |
| MyOne | 0.525 | 81 | 22 |
| M-280 | 1.415 | 54 | 108 |
| M-450 | 2.200 | 102 | 494 |

(1) Fonnum, G.; Johansson, C.; Molteberg, A.; Mørup, S.; Aksnes, E. Characterisation of Dynabeads® by Magnetization Measurements and Mössbauer Spectroscopy. *Journal of Magnetism and Magnetic Materials* **2005**, *293* (1), 41–47. https://doi.org/10.1016/J.JMMM.2005.01.041.

Table S4. Effect of the hybridization temperature, length of the biotinylated oligonucleotide and NaCL concentration in the Tris-HCL buffer on the capture efficiencies (%) of a fluorescently single-stranded DNA of 80 bases at 50 nM.

| **Temperature (°C)** | **Control^b^** | | | **NaCL concentration**  **20 bases** | | | **80 bases** | | |
| --- | --- | --- | --- | --- | --- | --- | --- | --- | --- |
|  | **100 mM** | **1 M** | | **100 mM** | **1 M** | | **100 mM** | **1 M** | |
| **49** | 10 ± 4.2^c^ | | 9 ± 4.6 | 18 ± 0.7 | | 44 ± 2.1 | 46 ± 3.5 | | 74 ± 1.5 |
| **59** | 7 ± 2.8 | | 8 ± 0.7 | 24 ± 2.8 | | 37 ± 8.5 | 54 ± 6.4 | | 63 ± 8.5 |

^a^ Capture efficiency (%) = *(I_0_ -I_C_ )I_0_×*100, I_0_ = fluorescence intensity of initial DNA solution, I_C_ = fluorescence intensity of the solution of non-captured DNA

^b^ Control corresponds to the experiments performed without beads functionalization

^c^ Data are presented as mean ± SD of duplicate


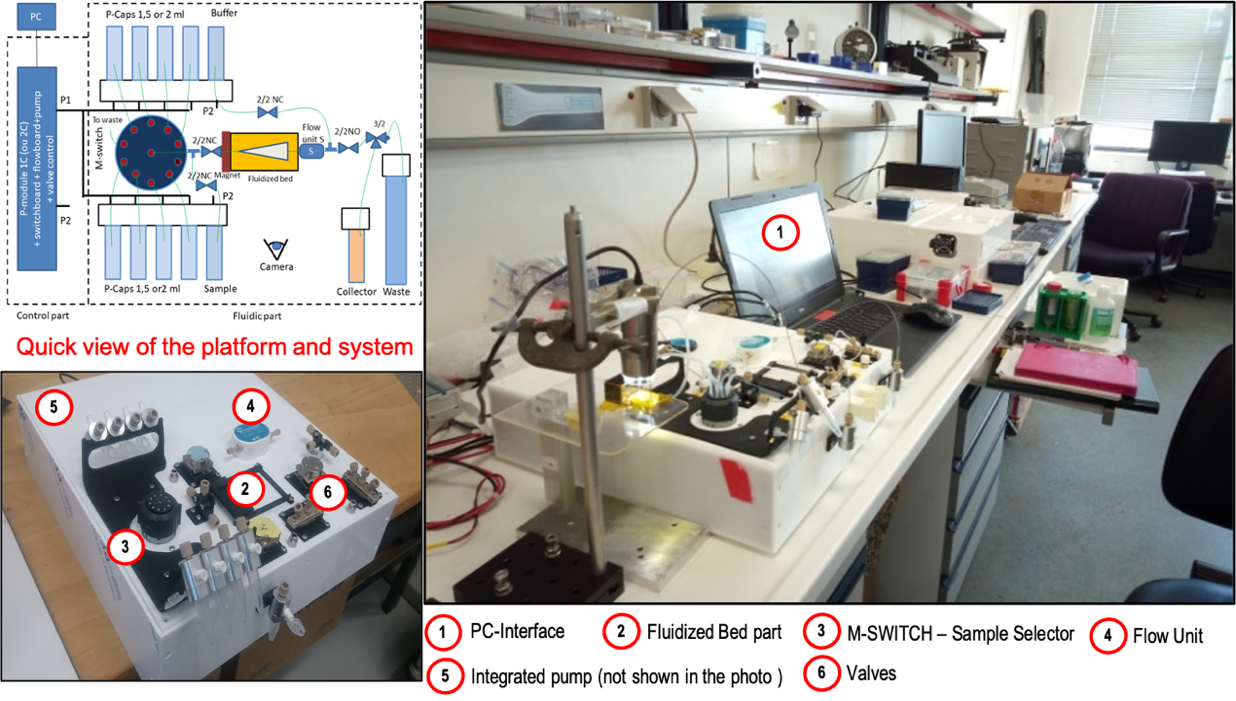


Figure S1. Schematic representation and pictures of the microfluidic platform for automated extraction on FB

Figure S2. Images of the fluidized bed at: A) 1 µL/min in a chip of 50 µm height, B) 1 µL/min in a chip of 250 µm height and C) 5 µL/min in a chip of 250 µm height.


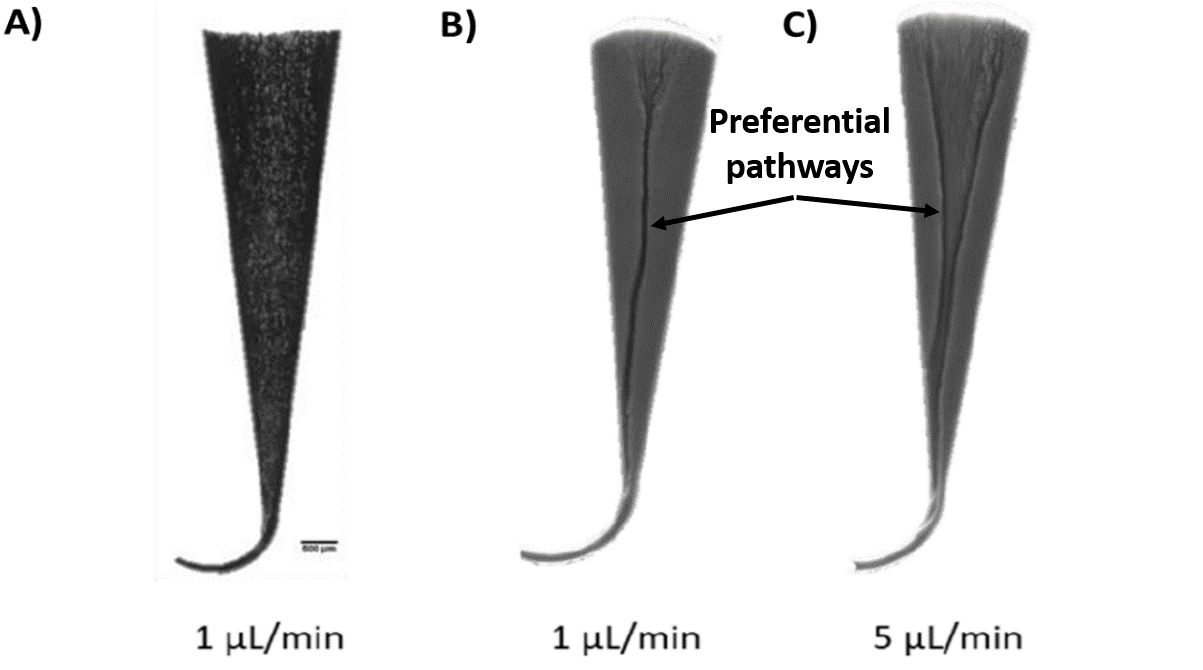

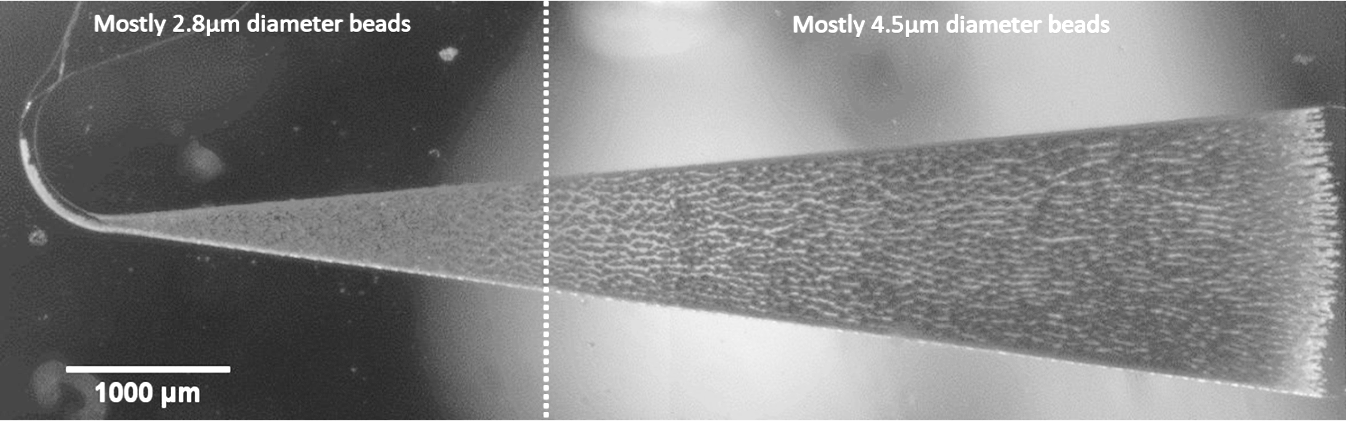


Figure S3. Images of bimodal matrix of beads at a flowrate of 5uL/min with segregation (75 % of 4.5μm diameter beads and 25 % 2.8μm diameter beads).

**
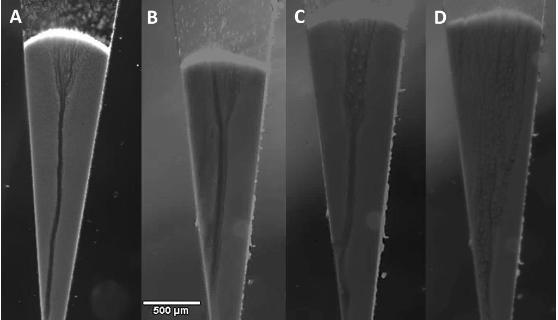
**

E


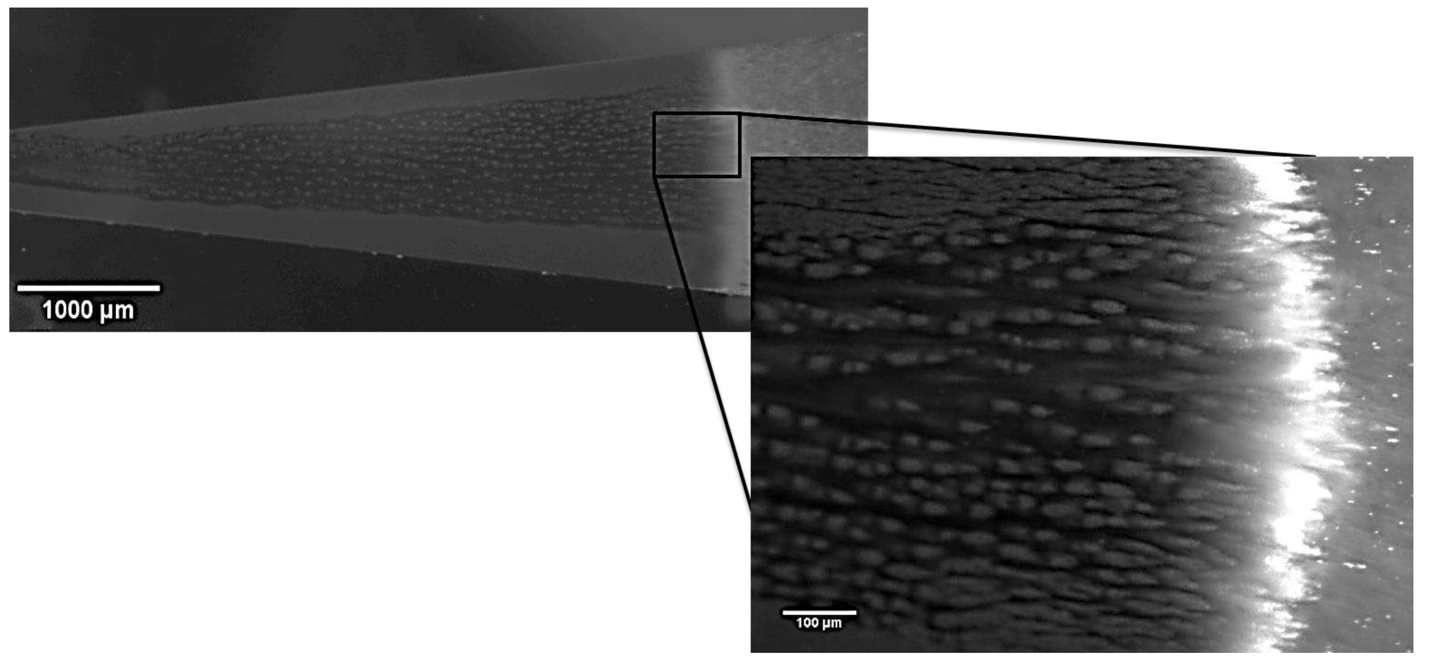


Figure S4. ﻿Pictures of the bed of particles in a 250 µm chip with different beads composition- M-270 COOH and A) + nothing B) + 4% of MyOne COOH, C)+ 20% of MyOne COOH and D) + 40% of MyOne COOH. Beads were dispersed in PBS and BSA 1%. Pictures were taken with a flow rate of 5µL/min (E) Images of bimodal matrix of beads (ratio 1:1) at a flowrate of 5uL/min without segregation (50 % of 1μm diameter beads and 50 % 2.8μm diameter beads).


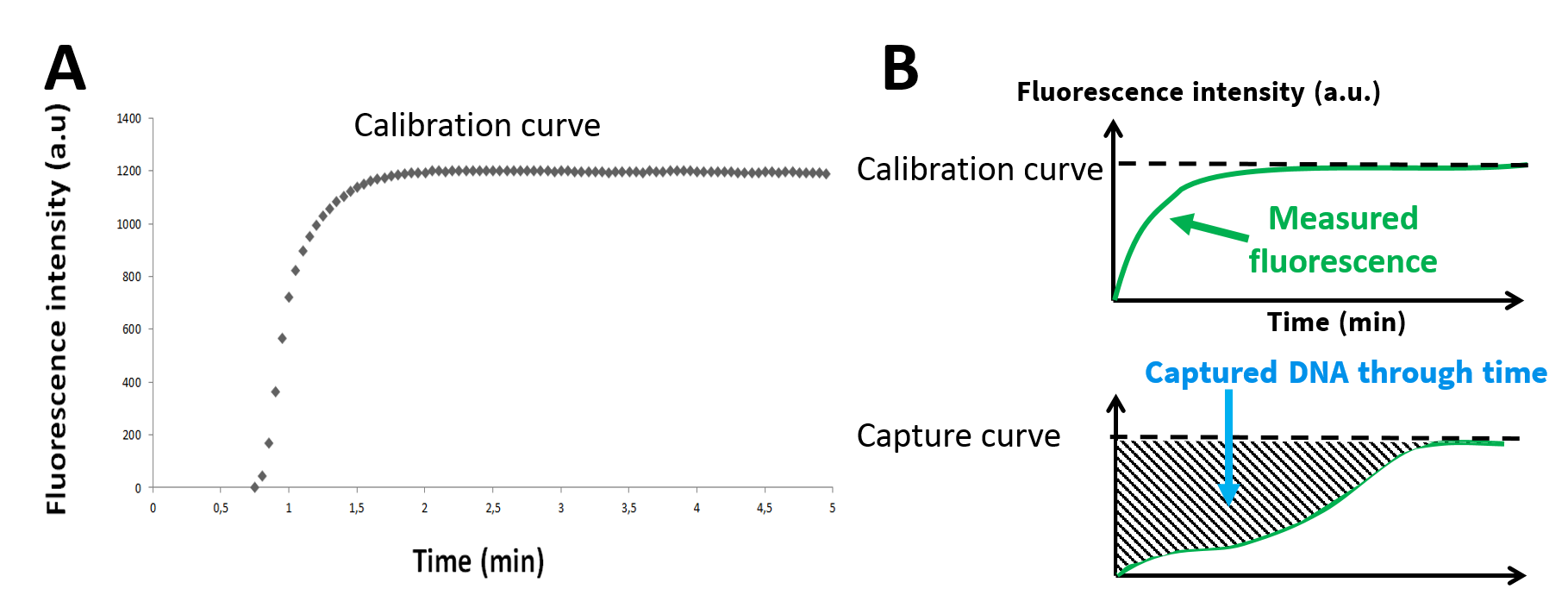


Figure S6. A) Calibration curve performed for extraction FB experiments showing fluorescence intensity in arbitrary unit (a.u) as a function of time in minutes (min). Fluorescent DNA sequence is spiked into the capturing buffer. Then the sample is injected inside the FB at the desired flow rate. The intensity is measured at the FB output channel. When the intensity reaches a plateau, the sample DNA concentration is correlated to that level of intensity. B) Schematic of calibration curve (top) and capture curve (bottom). The captured DNA is represented by the area between the intensity plateau and the measured fluorescence curve.


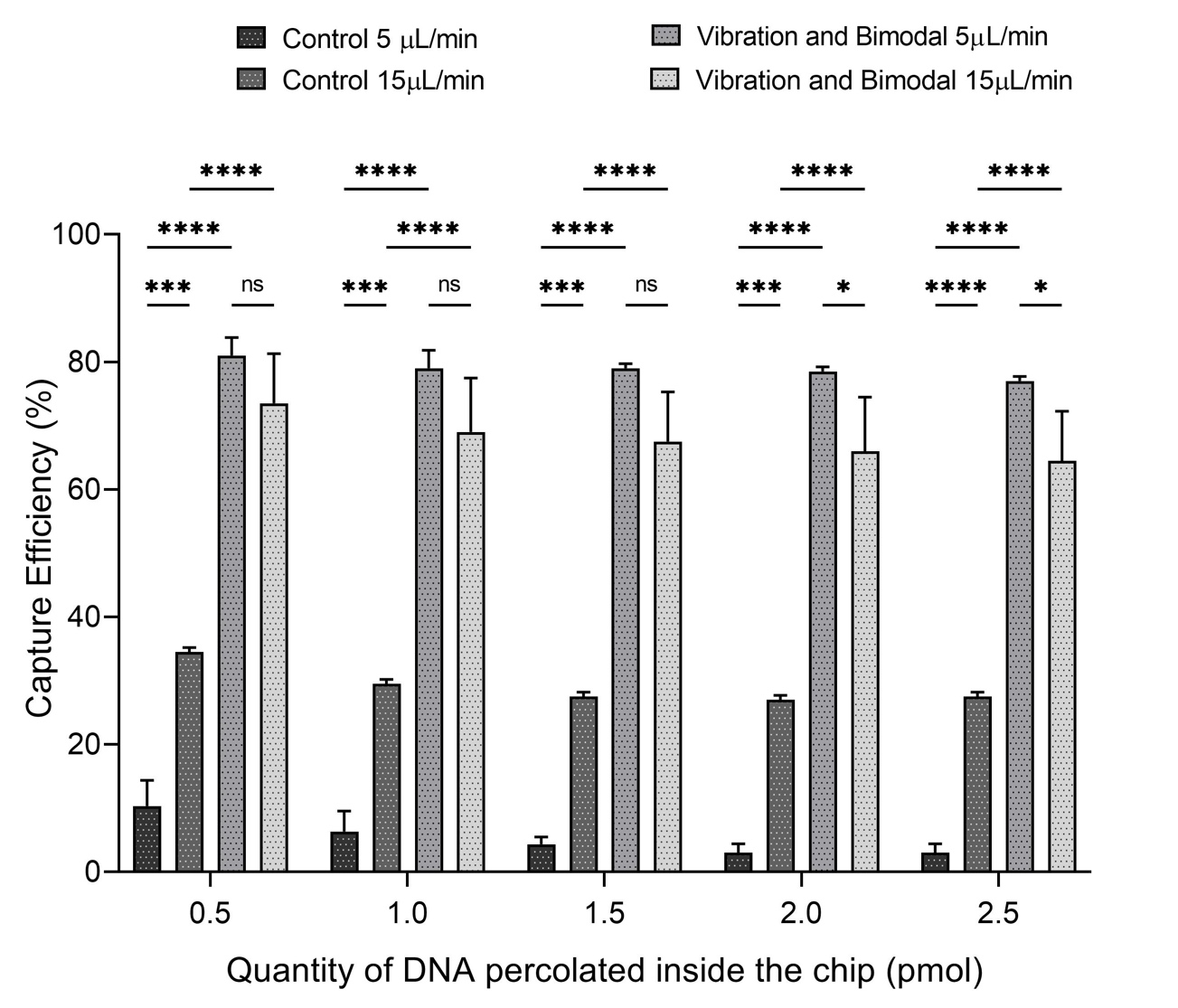


**Figure S5.** DNA capture efficiencies by hybridization on beads at 49°C as a function of the quantity of DNA percolated inside the FB (250 µm height) at 5 and 15 µl/min. The vibration and bimodal method is compared to the control. DNA was a fluorescent WT sequence (80 bases) of BRAF gene (****p <0.0001)


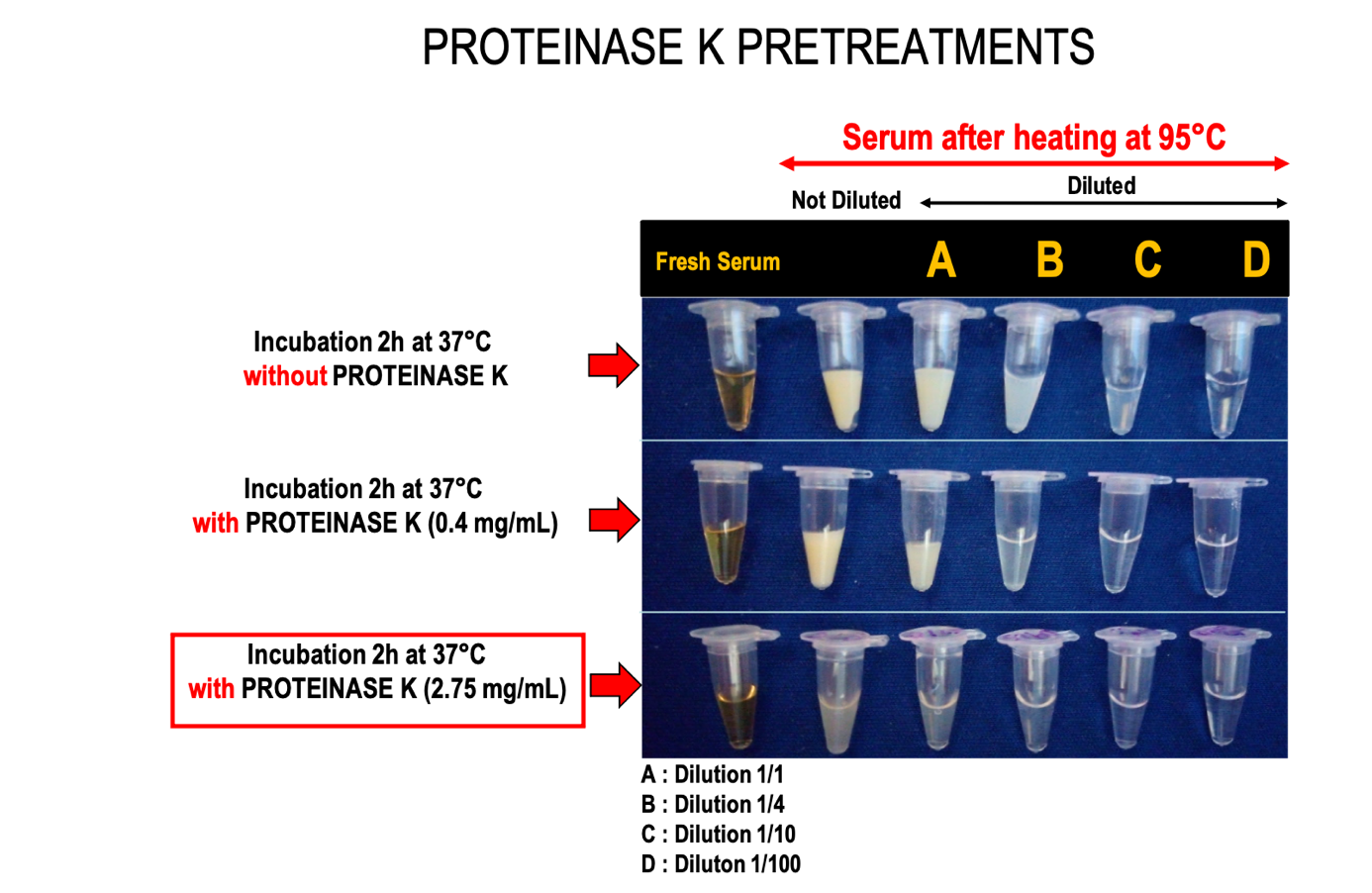


Figure S7. Images of serum diluted and undiluted pretreated with proteinase K at different concentrations (0-2.75 mg/mL). The best pretreatment conditions are shown in the red square.

# **Figure S8.** Schema of LCR amplification principle. It involves a thermostable ligase to join two probes which can be amplified by standard PCR cycling. Nucleotide T corresponds to the wild type sequence whereas A to the mutated one. For LCR, two pair of probes (Table 1S, K to N) are used fully complementary to the mutant (Mt) target but not to the wild type (Wt) sequence. The pair of probes (p1 and p2) is complementary to the sense strand of the *BRAF* sequence and the other (cp1 and cp2) to the antisense. The probes are also complementary to each other. Upon hybridization of the probes with the target, the ligase will fill the gap in the DNA strand between the probes by filling in the appropriate nucleotide. Ligase will only recognize and ligate nucleotides that properly anneal to the probe. The cycle can be repeated until the desired quantity of DNA is created.

**Chemicals and materials**

Tris-EDTA buffer solution (pH 8.0), Bovine Serum Albumin (BSA), phosphate-buffered saline (PBS), Tris(hydroxymethyl)aminomethane hydrochloride (Tris-HCl), Ethylenediaminetetraacetic acid (EDTA), sodium chloride(NaCl), biotin and KiCqStart SYBR^TM^ Green ready mix were obtained from Sigma-Aldrich (St-Louis, MO, USA). UltraPure^TM^ DNase/RNase-free distilled water (Invitrogen^TM^) and Proteinase K (20 mg/mL) (Invitrogen^TM^) were purchased from Thermofisher Scientific (Waltham, MA, USA). AmpLigase Thermostable DNA Ligase buffer was provided by Lucigen (Teddington, UK) and BSA (1mg/mL) by New England Biolabs (Ipswich, Massachussetts, USA). Poly (dimethylsiloxane) (PDMS) precursor (Sylgard 184 Silicone Elastomer) and curing agent were purchased from Dow Corning Corp. (Midland, MI, USA). Poly (dimethylacrylamide-co-allyl glycidyl ether) (PDMA-AGE) was provided by the Institute of Biocatalysis and Molecular Recognition (CNR) (Milan, Italy). All other chemical reagents were purchased from commercial sources in France and used as received.

Super-paramagnetic beads Dynabeads^TM^ M-270 Carboxylic acid (1 and 2.8 µm in diameter) and coated streptavidin beads such as Myone^TM^ Streptavidin T1 (1.0 µm in diameter) and M-280 Streptavidin (1 and 2.8 µm in diameter) were obtained from Thermofisher Scientific (Waltham, MA, USA). PEEK tubings 1/32” outer diameter (O.D) x 63 µm inner diameter (I.D), 1/32" O.D x 127µm I.D and 1/32" O.D x 250 µm I.D were obtained from Cluzeau Info Labo (Sainte-Foy-La-Grande, France). NdFeB12 permanent magnet (length 30 mm × height 20mm × width 20 mm) with magnetization through the width and a magnetic field intensity of 1.47T was provided by ChenYang Technologies (Finsing, Germany). These dimensions were chosen to provide a magnetic field oriented mainly along the channel axis and to minimize the lateral component of the magnetic forces.

**Washing and capturing buffers**

When needed, the magnetic beads were rinsed in washing (T2X) and (T1X) buffers (5 mM Tris-HCL pH 7.5, 0.5mM EDTA and 1M NaCl) as suggested by the manufacturer. The capturing buffer was composed of 10 mM Tris-HCL pH 7.5, 5mM EDTA, 1M NaCl and Tween 20 at 1% (w/v). Ultrapure Milli-Q® water (18.2 MΩ cm^−1^) (Direct-Q 3, Merck Millipore, Darmstadt, Germany) was used to prepare all buffers.

**Equipments**

A Micro Milling machine (Minitech Machinery Corp., Norcross, GA, USA) was used to fabricate brass micro-molds and a plasma cleaner system (Model Pico PCCE, Diener Electronic, Ebhausen, Germany) to oxidize the PDMS surface. An optical inverted epifluorescence microscope (Model Eclipse Ti-E, Nikon, Tokyo, Japan) equipped with a Nikon Intensilight C-HGFIE mercury lamp and CoolSNAP^TM^ HQ2 CCD camera (Teledyne Photometrics, Tucson, AZ, USA) was used to implement a microfluidic platform. ﻿The fluorescence detection was performed using 40x objective lens and green filters (excitation 455–490 nm and emission 505–555 nm). The collected fluorescence data were analyzed with NIS-Elements software (Nikon, Tokyo, Japan). Confocal Images were obtained on a Leica DMi8 microscope (Leica Microsystems GMbH, Weetzlar, Germany). When needed, the temperature was controlled means a glass slide coated with Indium Tin Oxide (ITO) with feedback regulation from a voltage controller (Eurotherm 3508, EU Automation, Stafford, UK) through a thermocouple placed inside the PDMS chip. A special reservoir block (reservoirs of 2 mL) was used to pressurize the samples (FLUIWELL-4C, Fluigent, Paris, France). The liquid flow control was achieved thanks to a pressure controller (from 1 to 1000 mbar) (FLOW EZ^TM^). A flowrate controller (FLOW UNIT, Fluigent, Paris, France) was used to precisely measure flow rate (Flow Unit S from 1 to 7µL/min and Flow Unit M from 1 to 80µL/min). An Eppendorf ThermoMixer^TM^ C (Eppendorf France SAS, Montesson, France) was used for beads preparation. A Biometra Thermocycler T-Gradient ThermoBlock (BiometraGmBH, Gottingen, Germany) was used to heat the samples during DNA extraction and a Cepheid^TM^ SmartCycler^TM^ Real-Time PCR detection system (Cepheid Inc, Sunnyvale, CA, USA) was used for qPCR analysis. LCR assays were carried out by using a FastGene^TM^ Ultracycler (Nippon Genetics Europe GmbH, Duren, Germany).

**Microfluidic FB platforms**

*Platform on Microscope.* A microfluidic platform was implemented on an optical epifluorescence microscope by assembling different modules. The pressure controller (Flow EZ) was connected to reservoir unit (Fluiwell-4C). PEEK tubings were used to connect the FB chip to the Fluiwell and to the other elements of the experimental setup. A 12 cm long PEEK tubing (63 µm I.D.) was placed at the entrance of the chip. The liquid flow inside the chip was produced by pressurization of the sample reservoir by using the pressure controller. A Flow Unit (M) was connected to the chip outlet allowed to regulate the flow rate and the pressure required for the experiments. Both pressure and flow rate were controlled with the software All-In-One (A-i-O) (Fluigent, Paris, France). A PEEK tubing (250 μm I.D.) was connected to the chip outlet and to the Flow Unit to direct the liquid either to a trash reservoir or to a collecting reservoir for subsequent analysis.

*Automated platform.* An automated microfluidic FB platform was also fabricated in this study. This automated prototype was developed by integrating different fluidic modules. It was fully controlled by a PC interface. The switchboard allowed the communication of the switches with the software while the flow board allowed to measure and regulate the flow. Pressure and flow rates required for the experiments were controlled with All-In-One (A-i-O) software (Fluigent, Paris, France). All the valves were controlled with ESS control software (Fluigent, Paris, France). A rotative valve (M-SWITCH) connected to 2 mL reservoirs allowed to sequentially inject the different buffers into the chip. The sample of interest could be separately injected into the chip via the use of pinch valves. These valves could be operated in ﻿a switch-on/switch-off mode. (Model 225 P12-11, NResearch^TM^ Inc, West Caldwell, NJ, USA). Thanks to this configuration, the time of arrival of the sample to the FB chamber was reduced and the risk of any contamination was avoided since the sample was not flowing through the M-SWITCH. The liquid flow was achieved by a pump integrated into the platform (not shown in the photo) with two independent pressure exits (P1 and P2). The flow rate was regulated at the exit of the chip by using a flow rate controller (Flow Unit S). A pinch valve placed at the chip output allowed to direct the liquid either to trash or to collecting reservoir. The inlet and outlet of the chip were connected to the PEEK tubing (1/32” O.D. x 0.127 mm I.D.). When needed, a digital and portative camera (Dino-Lite AM7013MZT Microscope USB, Dino-Lite, Taipei, Taiwan) was positioned on the top of the FB chamber.

**Microfluidic FB operating process: system preparation**

The equipment and microfluidic platforms are described in section S1.3. Before every experiment either by means of microscope or by using the automated platform, the whole system was initially filled with buffer PBS containing BSA (1%) (w/v). Afterwards, the injection of magnetic beads was performed by disconnecting the PEEK tubing at the chamber outlet and inserting a pipette tip which was half-filled with PBS-BSA buffer. The magnetic beads were loaded inside the tip and guided at the right location in the chamber by using a small magnet. Finally, the NdFeB12 permanent magnet was placed close to the chip inlet and aligned with the chamber axis at a 1.50 mm distance. Once both beads and magnet were in place, fluids were ready to flow through the system.

**Optimization of capture conditions**

To optimize the capture conditions, Dynabeads^TM^ MyOne Streptavidin T1 (250 µg) were functionalized of 20 or 80 bases (Table S1, E and F) complementary to target fluorescent DNA. First, 5 µl of beads stock solution at 10 mg/mL (50 µg) were placed in an Eppendorf tube washed three times with 2X buffer (50 μL) and then resuspended in 100 µL of capture probes prepared at 1 µM. Next, the beads were then incubated in a thermomixer at 20°C for 50 min at 800 rpm, washed 3 times and resuspended in 100 μL of 1X buffer. Afterwards, the functionalized beads were placed in a magnetic rack, the buffer was discarded and the beads were mixed with 50 µL of fluorescent WT DNA (Table S1, B) solution prepared at 50 nM in Tris-HCl buffer at two NaCl concentrations of: 100 nM or 1M. The tubes were then incubated in the thermomixer at 49 and 59°C for 50 min at 800 rpm for the hybridization of DNA. Control experiments were also performed by using non-functionalized beads. After capture, the beads were placed on a magnetic rack and the supernatant was recovered and immediately injected in fluidized bed for fluorescent detection (50 µm in height) at 2 µl/min in absence of beads. This fluorescence intensity was considered as the one of non-captured DNA on beads and it was compared to the initial DNA solution previously injected in the chip at the same flow rate during around 5 min. The fluorescence intensity of the initial DNA solution was recorded at the chip output channel and was used as calibration measurement (Figure S4).

**Fabrication of microchips**

The 2^nd^ generation microfluidic fluidized bed chips were fabricated by standard casting of PDMS on prefabricated micro-milled molds. These molds were micro-milled in a brass piece (5 cm x 5cm). ﻿Sylgard 184 PDMS prepolymer and curing agent were mixed at a ratio of 10 :1 (w/w). After blending, the mixture was degassed in a vacuum dessicator for 1h. ﻿Then, the PDMS pre-polymer mixture was poured onto the molds. A second flat PDMS layer of a 1mm (in height) was prepared in a petri dish by pouring 13 g of the PDMS mixture. Both parts were then cured in an oven at 73°C for 3h. The cured PDMS cap was peeled off from the mold and cut into suitable shape. The chamber inlet/outlet were drilled using a puncher tool (0.75 mm). The cured cap and flat PDMS layer were then oxidized in the plasma cleaner (40 mbar, 30 sec, 200W) and immediately laid on top of each other for an irreversible bonding. To finish, a surface treatment with PDMA-AGE (0.5% w/v) was performed by incubating the solution inside the chip at room temperature (RT) for 2h followed by a rinsing with Milli-Q® water and drying with compressed air.

**Serum samples**

Blood samples were obtained from healthy donors (Etablissement Français du Sang (EFS), France) and let at room temperature to allow the blood clotting. The clot was then removed by centrifugation at 2500 x *g* in a refrigerated centrifuge and the resulting supernatant designed as “*serum*” was recovered. The serum samples were then aliquoted and stored at -20ºC. A new aliquot was thawed at 4ºC before each experiment.
